# Enhancing the nitrogen removal from swine wastewater digested liquid in a trickling biofilter with a soil layer

**DOI:** 10.1039/d0ra03333b

**Published:** 2020-06-23

**Authors:** Bowei Zhao, Fei Xie, Xiao Zhang, Xiuping Yue

**Affiliations:** College of Environmental Science and Engineering, Taiyuan University of Technology 79 Yingzexi Road Taiyuan 030000 P. R. China yuexiuping@tyut.edu.cn +86 0351-3176586

## Abstract

Trickling biofilters (TFs) allow for a simultaneous nitrification and denitrification (SND) process, and offer a favorable solution for the treatment of swine-wastewater digested liquid due to their simple operation and low cost. In this study, a soil trickling biofilter (STF) was developed to enhance nitrogen removal. A gravel trickling filter (GTF) and a woodchip trickling filter (WTF) were also constructed and operated synchronously to demonstrate the advantage of micron-sized media. The results showed that the STF had a higher ammonium nitrogen (NH_4_^+^-N) removal capacity of 21.4%, 24.9%, and 18.3% in comparison to the GTF when the influent NH_4_^+^-N was 192.9 mg L^−1^, 500.2 mg L^−1^ and 802.1 mg L^−1^, respectively. The total nitrogen (TN) removal capacity of the STF was 104.6%, 89.4%, and 37.5% higher than that of the WTF. Thus, the addition of micron-sized soil to TF could increase the systemic nitrogen removal capacity.

## Abstract 2

Trickling biofilters (TFs) allow for a simultaneous nitrification and denitrification (SND) process, and offer a favorable solution for the treatment of swine-wastewater digested liquid due to their simple operation and low cost.

## Graphical abstract

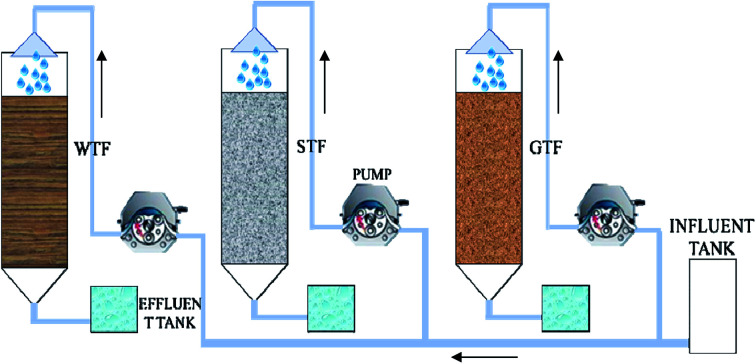

## 1. Introduction

Swine wastewater from large-scale farms can have a high chemical oxygen demand (COD) and high ammonium nitrogen (NH_4_^+^-N) concentration, thus making it a major pollutant in vast rural areas.^1^ Swine wastewater that is not properly disposed of can cause serious problems in the agricultural environment, for example, eutrophication and zoonosis.^2–4^ With respect to the economy, environmental sustainability, and social acceptability, the technologies for swine wastewater treatment should be low-cost, energy-saving, low-maintenance, effective, and stable.^5,6^

The key point of swine wastewater treatment is the removal of nitrogen from the digested liquid with a low carbon/nitrogen ratio (C/N).^7^ Most technologies for nitrogen removal are based on the conventional nitrification and denitrification processes. Generally, the traditional biological nitrogen removal process includes plug flow reactors [*e.g.*, anoxic oxic (A/O)]^8^ and continuous stirred tank reactors [*e.g.*, sequencing batch reactor (SBR)].^9,10^ Although these techniques have a favorable effect on nitrogen removal during swine-wastewater digested liquid treatment, they are disadvantageous in terms of their running costs.

The modern nitrogen removal processes include single high ammonia removal over nitrite (SHARON), anaerobic ammonium oxidation (ANAMMOX), complete autotrophic nitrogen removal over nitrite (CANON), and oxygen-limited autotrophic nitrification–denitrification (OLAND). These methods have several advantages in terms of their low running costs due to the money saved for aeration, the organic carbon source for denitrification, and the reactor volume.^11–13^ Unfortunately, these technologies require strict operational conditions to maintain the stability of the system, which increases the operation difficulties and restrict their widespread application.

Trickling biofilter (TF) is a biofilm wastewater treatment process that has been generally recognized as providing an effective simultaneous nitrification and denitrification (SND) process with a low-cost and convenient management. TF has been widely used to treat domestic wastewater, piggery wastewater, textile wastewater, and leachate.^14^ The biochemical process is the main pollutant removal pathway, whereby the microbes growing on the surface of the filter material play the major role.^15^ Hence, the characteristics of the filter material are key factors affecting the efficiency of TF.

An excellent filter material should have a large specific surface area and a good biofilm adhesion. Various materials have been investigated as filter material for use in TF systems, for example, zeolite, sand, plastic, woodchip and complex material.^16^ This study focused on the surface characteristics of the filter media to obtain favorable treatment efficiency. With the exception of the surface characteristics, the size of the filter material is the most direct factor affecting the specific surface area of the material. In principle, the smaller the size of a filter material, the larger specific surface area is for the enrichment of microbes. Soil, as the natively weathered gravel, generally has a small size between millimeters and microns. Hence, soil infiltration technology has long since been used for water treatment.^17,18^ However, the clogging and blocking of the soil layer limit the treatment capacity of soil infiltration, and also prevent the utilization of soil in TF systems.^1^

In this study, a novel soil TF (STF) is proposed to solve the clogging problem of a micron-sized soil filter material under a high hydraulic and organic load. This novel technique involves the micron-sized soil adhering to a woodchip framework. Thus, the soil layer that becomes fixed to the framework can resist a certain hydraulic load and has a large specific surface area to adhere to the biofilm. In order to compare the advantages of this STF process, a gravel TF (GTF) and a woodchip TF (WTF) were also constructed and synchronously operated. The aim of using GTF is to demonstrate the size advantage of the micron-size soil for the enrichment of microbes, whereas the aim of using the WTF is to show the effect of the woodchip framework in the STF.

## 2. Materials and methods

### 2.1 Wastewater

Raw wastewater was collected from a local pig farm. The influent used in this study was the digested liquid with a low C/N ratio from the UASB treatment of the raw wastewater. The characteristics of the digested liquid are listed in Table 1.

**Table 1:** Characteristics of swine-wastewater digested liquid

Period	Day	COD (mg L^−1^)	NH_4_^+^-N (mg L^−1^)	TKN (mg L^−1^)	TN (mg L^−1^)	TP (mg L^−1^)	pH
Startup	1–55	295.1	489.7	492.8	496.6	18.7	8.3
I	56–155	180.9	192.9	194.5	198.1	8.7	8.0
II	156–255	293.1	500.2	502.2	503.8	19.3	8.3
III	256–346	508.9	802.1	804.5	807.3	29.2	8.5

### 2.2 Reactors

**Fig. 1:**
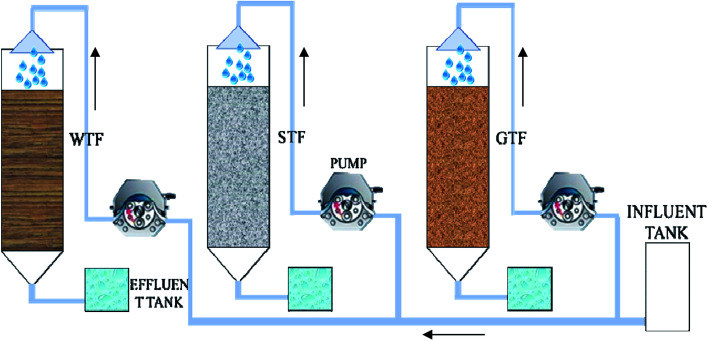
Schematic diagram of three trickling filter reactors.

Three TF reactors of the same size were constructed, each with a 0.3 m high plexiglass column of 0.1 m diameter (Fig. 1). The total volume of each reactor was 2.0 L, with an effective volume of 1.5 L. A circular cone with a volume of 0.2 L was attached to the bottom of the plexiglass column. There were two symmetrical vents with an area of 2.5 cm^2^ between the filter bed and the circular cone to allow natural ventilation.

### 2.3 Filter material

In this study, pine wood was chosen to make the woodchips, which were 3–5 cm high, 2–3 cm wide, and 0.5–0.8 cm thick. Gravel with a diameter of 1 cm was used for the filter material in the GTF. The soil was ground to a powder and sieved through a screen of 160 meshes per square centimeter. The bulk density, porosity, and ash content of the soil were 0.855 g cm^−3^, 50% (v/v), and 81.12% (w/w), respectively. Woodchips (of the same size as those used as the filter material in the WTF) provided the framework in the STF.

To create a soil layer within the framework of the STF, the woodchips were soaked in fresh water and then rolled over the prepared dry soil powder. As the woodchips were damp, the soil adhered onto the surface of the woodchips. The bulk volume ratio of the woodchips to the soil in the STF was approximately 2 : 1. To compare the efficiency of the three TFs, the void volume ratio for each of the three TFs was about 50%.

### 2.4 Operation

The three TFs were fed with the same swine wastewater digested liquid. The wastewater was evenly sprayed onto the top of the filter bed with a hydraulic loading of 0.17 m^3^ m^−3^ d^−1^ during all operating periods. The effluent was collected by the cone and discharged from the bottom. There were no inoculums with microorganisms for all reactors before starting-up. There were four operating periods according to the different organic volume loading rate (OLR) and influent quality. All three reactors used natural aeration and were operated at an ambient temperature of between 15 °C and 25 °C. The operational parameters are illustrated in Table 1.

### 2.5 Wastewater quality analysis

The COD, NH_4_^+^-N, nitrite nitrogen (NO_2_^−^-N), nitrate nitrogen (NO_3_^−^-N), and total nitrogen (TN) were analyzed according to the standard methods for the examination of water and wastewater.^7^ The concentrations were determined using a spectrophotometer (Shimadzu, UV2500). The pH was monitored by a DELTA 320 pH meter. Normally, the influent and effluent were sampled once every 5 days for analysis.

### 2.6 Observation of the filter media

Before operating the reactors, the woodchips, gravel, soil, and woodchips wrapped with soil were all sampled. The samples were soaked in a 2.5% glutaraldehyde solution (pH 7.2) for 2 h. Then, all samples were washed three times with a 0.1 mol L^−1^ phosphate buffer (pH 7.2). Alcohol at a concentration of 50%, 60%, 70%, 80%, 90%, and 100% was in turn used to dehydrate the samples, whereby *tert*-butyl alcohol gradually replaced the alcohol. Afterward, the samples were dried by a desiccator (HCP-2, HITACHI). Finally, the samples were plated with a gold film of 1 nm by an ion sputtering coater (E-1010, HITACHI) and observed with a scanning electron microscope (SEM).

### 2.7 Analyses using PCR and DGGE

On the last day of operation, filter material was sampled from the three TFs. Genomic DNA of all samples was extracted using DNA isolation kit components (MOBIO), which was performed according to the manufacturer's operations manual. The purity and quantity of the extracted DNA were determined by ultraviolet spectrophotometry at 260 nm and 280 nm, and the DNA was subsequently stored at −20 °C. A nested polymerase chain reaction (PCR) was used to amplify the bacterial specific 16S rRNA gene for denaturing gradient gel electrophoresis (DGGE). The bacterial specific primers CTO189f and CTO654r were used in the PCR to amplify the DNA fragments. The specific procedures of the PCR and DGGE analyses were conducted according to.^7^

## 3. Results

### 3.1 Observation of filter media before operation

**Fig. 2:**
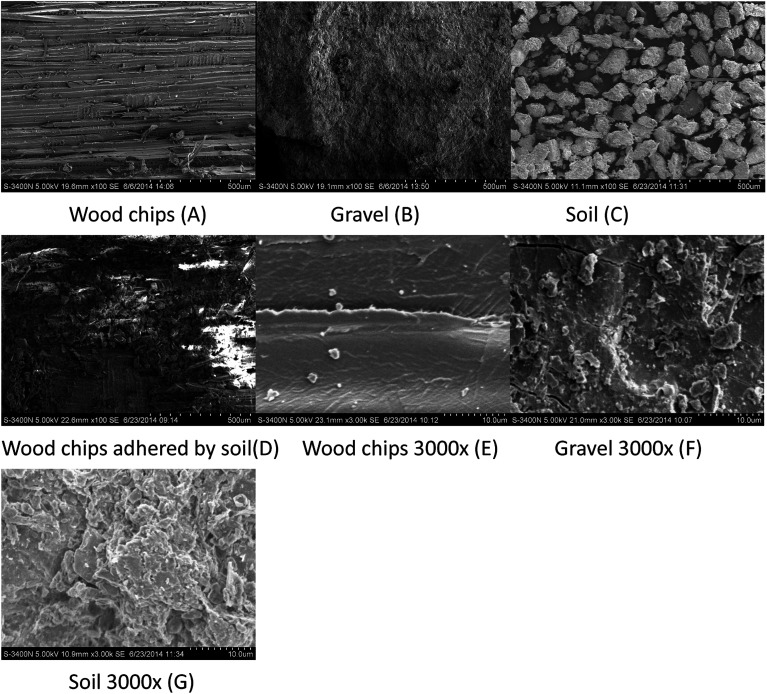
SEM images of the filter media before operation: (A) wood chips; (B) gravel; (C) soil; (D) wood chips adhered by soil; (E) wood chips 3000×; (F) gravel 3000×; (G) soil 3000×.

Fig. 2A–D shows the surface of the woodchips, gravel, micron-sized soil, and soil with woodchips in magnified SEM images (×100). As can be seen in Fig. 2A and B, some cut stripes were clear on the woodchips and a crude surface was observed on the gravel. As Fig. 2C shows, the size of a soil granule ranged between 50 and 100 μm, which indicates that, after grinding and screening through the 160 mesh, the natural soil could easily maintain a micron-size. The woodchips that were wrapped with soil are illustrated in Fig. 2D, which clearly displays a thin layer of micron-sized soil adhering to the woodchips. The cut stripes on the surface of the woodchips had been completely covered by the micron-sized soil. Moreover, Fig. 2E–G reveal the surface details of the woodchips, gravel, and micron-sized soil in magnified SEM images (×3000). In particular, Fig. 2E illustrates the smooth wood surface and the side of the wood fiber layer. Comparing Fig. 2F and G, the surfaces of the soil and gravel were similar and both were crude; hence, the main difference between the soil and gravel was their size due to natural weathering and our operation. The pores on the woodchip surface were barely noticeable (Fig. 2A and E), even when magnified ×3000. The pore size of the woodchips with soil adhered (inferred as being 8–10 μm from Fig. 2D) was significantly smaller than that of the gravel and soil only, which contributed to its larger specific surface area for microorganisms living.

### 3.2 Removal of COD

**Fig. 3:**
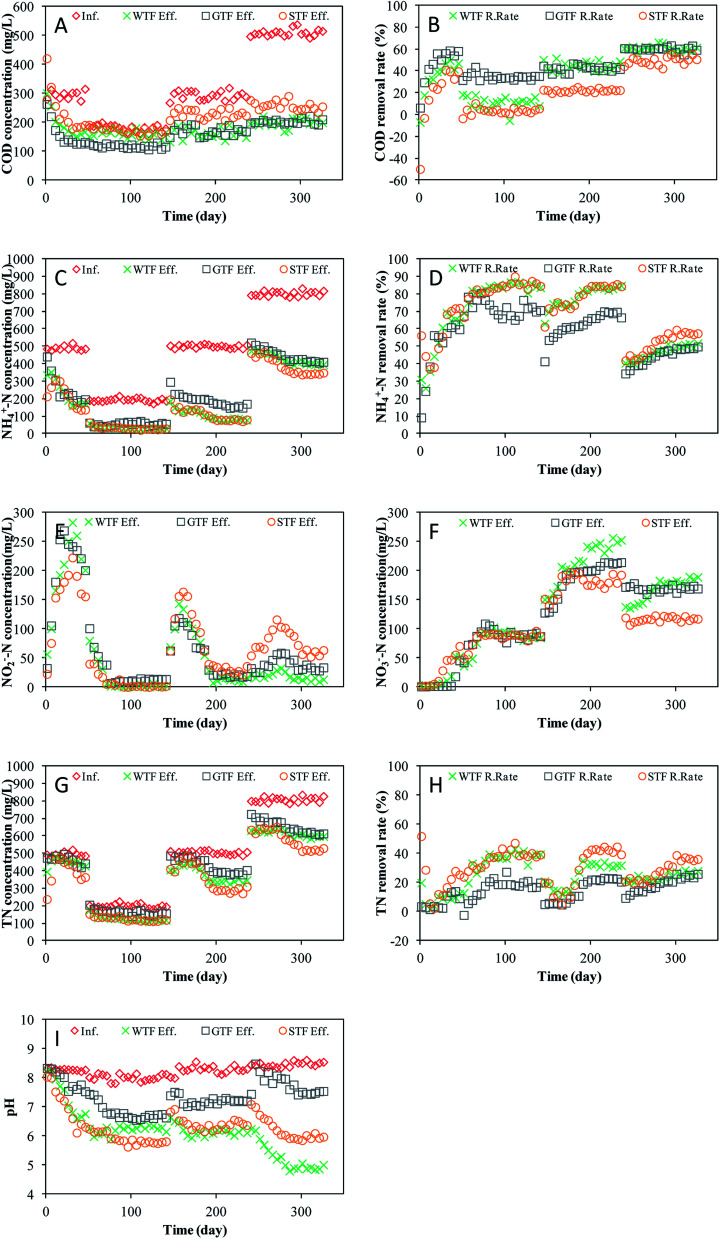
The performance of the three TFs: (A) COD concentration; (B) COD removal rate; (C) NH_4_^+^-N concentration; (D) NH_4_^+^-N removal rate; (E) NO_2_^−^-N concentration; (F) NO_3_^−^-N concentration; (G) TN concentration; (H) TN removal rate; (I) pH value.

The three TF reactors were started at a hydraulic loading volume of 0.17 m^3^ m^−3^ d^−1^ with an organic volume loading rate (OLR) of 50.8 g m^−3^ d^−1^ COD. The removal performances for the COD in the three TFs are presented in Fig. 3A and B. During the first 30 days, the COD of the effluent from each of the three TFs all reduced rapidly. After day 30, the COD stabilized. From day 30 to the end of start-up period, the average COD removal efficiencies of the WTF, GTF, and STF were 46.38%, 56.77%, and 38.28%, respectively. Moreover, the COD in the effluent in the STF was higher than that in the influent during the first 5 days because some intrinsic organic matter in the soil was removed by the effluent.

The influent COD during period I, II, and III was 180.9 mg L^−1^, 293.1 mg L^−1^, and 508.9 mg L^−1^ with an organic load of 30.8 g m^−3^ d^−1^, 49.8 g m^−3^ d^−1^, and 86.5 g m^−3^ d^−1^, respectively (Fig. 3A). The COD removal efficiencies of all three TFs recovered and were stable again within 5 days once the influent changed. This indicates that the TF process in all of the reactors had a good capacity to resist the organic load impact, which agrees with previous studies.^19,20^ The average COD removal rate of the WTF during the stable stages of periods I, II, and III (*i.e.*, day 56 to day 155, day 156 to day 255, and day 256 to day 346, respectively) was 11.0%, 44.2%, and 60.6%, respectively. Meanwhile, the COD removal rate of the GTF during the stable stages of periods I, II, and III was 35.1%, 41.7%, and 60.1%, and that of the STF was 3.9%, 21.7%, and 49.6%, respectively (Fig. 3B).

### 3.3 Ammonium-nitrogen removal and nitrification

The NH_4_^+^-N concentration and removal rate of the three TFs are displayed in Fig. 3C and D. During the first 15 days, the WTF had a low but stable NH_4_^+^-N removal rate, which increased gradually until day 30 before stabilizing at 65.89%. The NH_4_^+^-N removal of the GTF increased rapidly during the first 15 days and then slowly after day 16. At the stable state of the start-up period, the NH_4_^+^-N removal rate of the GTF fluctuated at ∼61.63%. The NH_4_^+^-N removal rate of the STF exhibited a “V” trend with the lowest point being on day 10. The NH_4_^+^-N removal rate was stable at 72.32% after day 40. Dissolved oxygen (DO) was used to oxidize ammonia, either by ammonia oxidizing bacteria (AOB) or nitrite oxidizing bacteria (NOB). The moisture content of the STF was higher than that of the WTF and GTF; therefore, the DO concentration in the solution of the STF was relatively high. As shown in Fig. 3D and E, the NH_4_^+^-N removal rate was highest in the STF, nevertheless, the NO_2_^−^-N concentration in the effluent of the STF was also the highest of three TFs. This indicates that nitrogen removal by AOB was higher than that by NOB.

The NO_2_^−^-N and NO_3_^−^-N concentrations in the effluents of the three TFs are shown in Fig. 3E and F. All three TFs had a similar variation in the NO_2_^−^-N concentration, which first increased and then decreased. When the effluent NO_2_-N concentration reduced, the effluent NO_3_^−^-N concentration increased, which agrees with the general rule of bio-nitrification. The highest effluent NO_2_-N concentration in the WTF, GTF, and STF occurred on day 21 (282.8 mg L^−1^), day 31 (268.6 mg L^−1^), and day 31 (222.4 mg L^−1^), respectively. Towards the end of the start-up period, the effluent NO_3_^−^-N concentration of the WTF, GTF, and STF was 52.6 mg L^−1^, 45.6 mg L^−1^, and 70.5 mg L^−1^, whereas the effluent NO_2_^−^-N decreased to 200.9 mg L^−1^, 200.5 mg L^−1^, and 155.6 mg L^−1^.

There were two main processes involved in the removal of NH_4_^+^-N: physiochemical and microbe catalysis. During the start-up period, the abundance of microbes was lower with a relatively low activity; thus, absorption to the filter media was the main process of NH_4_^+^-N removal. Although Buelna^21^ considered that NH_4_^+^-N stripping was a main removal process at high pH, absorption may have been more important in the start-up period of TFs in this study. Different trends were evident when comparing the NH_4_^+^-N removal in the WTF, GTF, and STF, which could have been caused by adsorption to the filter material. Due to the high adsorption to soil, the STF had a relatively high NH_4_^+^-N removal during the first 10 days.

During period I, the average influent NH_4_^+^-N concentration was 192.9 mg L^−1^ with an average nitrogen loading ratio (NLR) of 32.79 g m^−3^ d^−1^. The NH_4_^+^-N removal rate of the three TFs increased gradually (Fig. 3C) and the effluent NO_2_^−^-N concentration reduced sharply as the NO_3_^−^-N concentration increased (Fig. 3E and F). All three reactors subsequently entered a stable stage from day 81 to day 151, during which, the NH_4_^+^-N removal rate of the WTF, GTF, and STF was 84.50%, 69.80%, and 84.86%, respectively. The average NO_2_-N concentration in the effluents of the WTF, GTF, and STF during this period was 2.1 mg L^−1^, 11.7 mg L^−1^, and 2.5 mg L^−1^, respectively, whereas the NO_3_^−^-N concentration was 90.6 mg L^−1^, 90.0 mg L^−1^, and 88.4 mg L^−1^, respectively. The average NH_4_^+^-N concentration in the influent increased to 500.2 mg L^−1^ during period II, while the NLR was 85.03 g m^−3^ d^−1^. The NH_4_^+^-N removal rates of the three TFs reduced sharply during period II before recovering in following 45 days. From day 206 to day 251, the amount of NH_4_^+^-N removed by the WTF, GTF, and STF was maintained at approximately 186.2 mg L^−1^, 295.6 mg L^−1^, and 195.7 mg L^−1^, respectively. The NO_2_^−^-N concentration in the effluents of the three TFs increased in early stage and then reduced. During the stable stage of period II, the average NO_2_-N concentration in the effluents of the WTF, GTF, and STF was 14.1 mg L^−1^, 21.1 mg L^−1^, and 31.0 mg L^−1^, respectively. The NO_3_^−^-N concentration in the effluents of the WTF, GTF, and STF all increased gradually before stabilizing after day 206 at approximately 241.4 mg L^−1^, 207.3 mg L^−1^, and 181.0 mg L^−1^.

When the influent NH_4_^+^-N concentration was further increased during period III, the average NLR was 136.35 g m^−3^ d^−1^, and the NH_4_^+^-N removal rate of the three TFs was further lowered. When stabilized, the average NH_4_^+^-N removal rate of the WTF, GTF, and STF was 50.13%, 48.44%, and 57.32%, respectively. After day 291, the NO_2_^−^-N concentrations in the effluents of the WTF, GTF, and STF were stable at approximately 32.5 mg L^−1^, 58.8 mg L^−1^, and 116.1 mg L^−1^. The NO_3_^−^-N concentration in the effluents of the GTF, STF, and WTF fluctuated slightly around the average values of 169.3 mg L^−1^, 116.8 mg L^−1^, and 182.2 mg L^−1^, respectively.

### 3.4 Total nitrogen removal and denitrification

The three TFs commenced with a TN volume loading of ∼84.43 g m^−3^ d^−1^. The TN concentrations of the influents and effluents in each TF are shown in Fig. 3G and H. During the first 15 days, the WTF and STF had a certain TN removal, whereas the GTF did not. The TN removal of the STF and WTF should be given by the adsorption of ammonia to each filter material. After day 21, the TN removal rate of the three TFs increased gradually until day 46 when the TN removal rate of the WTF, GTF, and STF was 12.23%, 9.05%, and 25.32%, respectively. As the influent concentration increased, the TN volume loading was 33.67 g m^−3^ d^−1^, 85.64 g m^−3^ d^−1^, and 137.25 g m^−3^ d^−1^ during periods I, II, and III, respectively. After each change of the influent, the TN removal rate of the three TFs synchronously recovered and stabilized. During the stable stages of period I, II, and III, the average TN removal efficiency of the WTF was 38.08%, 32.64%, and 26.05%, respectively, whereas that of the GTF was 19.23%, 22.06%, and 23.81%, respectively, and that of the STF was 39.36%, 41.85%, and 35.85%, respectively.

**Fig. 4:**
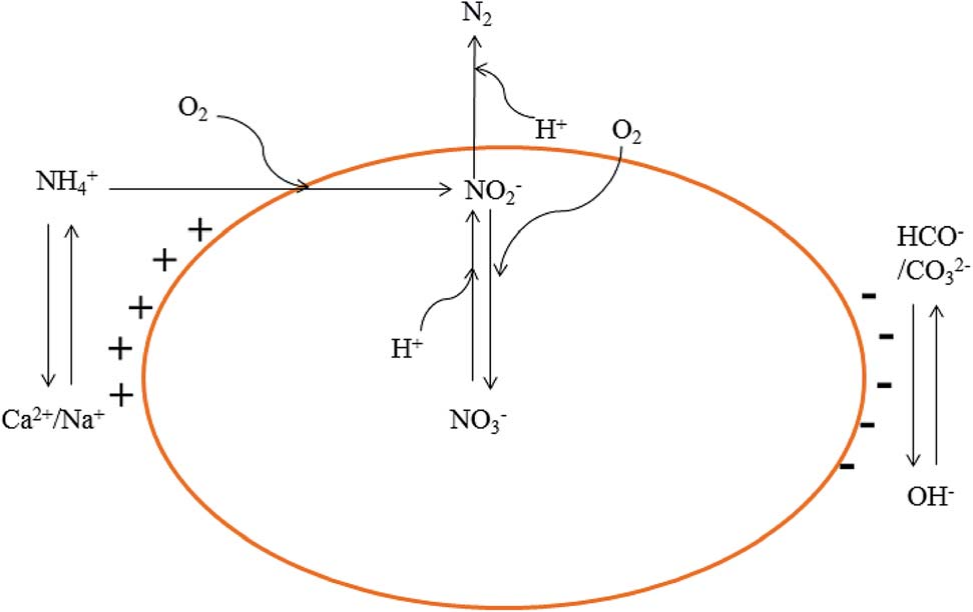
Ions transfer in the reactions.

As shown in Fig. 4, simultaneous nitrification and denitrification occurred in the three reactors, accounting for the nitrogen removal. The Ca^2+^ and Na^+^ adsorbed to the surface of the soil was replaced by NH_4_^+^, and the Ca^2+^ and Na^+^ precipitated after combining with OH^−^. The cations associated with OH^−^, HCO^−^, and CO_3_^2−^ anions form a dynamic balance of alternating ion changes. In the AOB process, oxygen is consumed for the oxidization of ammonia to nitrite. In addition, H^+^ ions are generated from this reaction (eqn (1)). The nitrite and nitrate that are produced are reduced to N_2_ during the denitrification process while H^+^ ions are consumed (eqn (2)):^22^

1 NH_4_^+^ + 1.24O_2_ + 0.16CO_2_ + 0.04HCO_3_^−^ → 0.04C_5_H_7_O_2_N + 0.96NO_2_^−^ + 0.94H_2_O + 1.9H^+^

2 0.1561NO_3_^−^ + 0.1167CH_3_OH + 0.1561H^+^ → 0.0095C_5_H_7_O_2_N + 0.119CO_2_ + 0.3781H_2_O

### 3.5 Analysis of AOB by PCR–DGGE

**Fig. 5:**
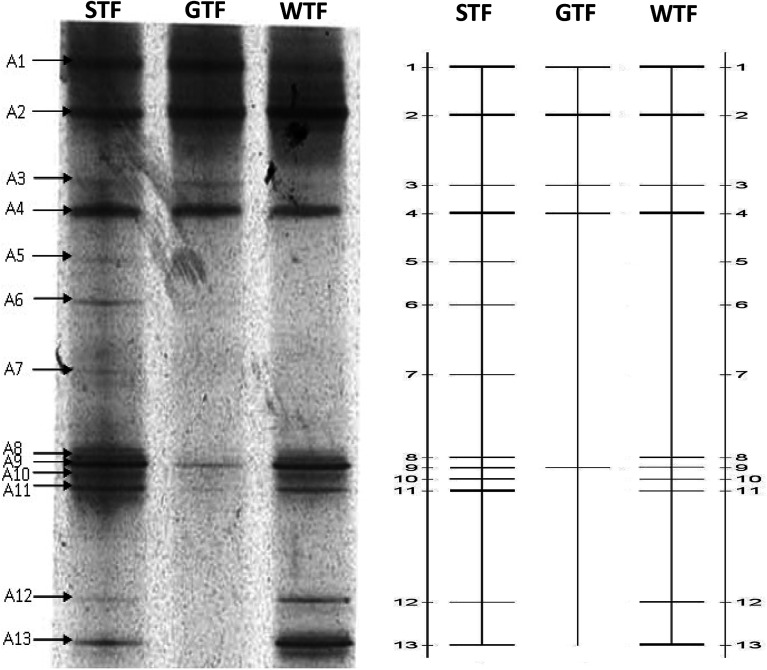
Analysis of AOBs using PCR–DGGE.

**Fig. 6:**
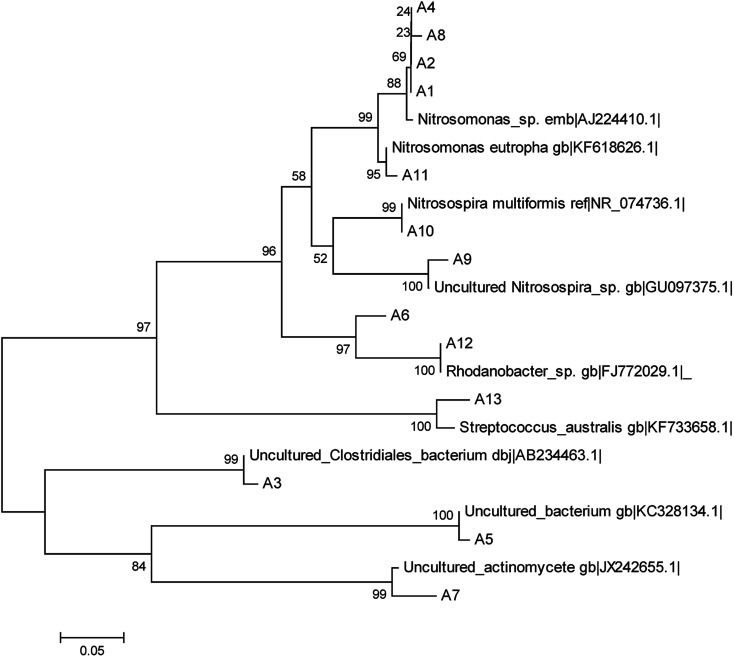
Neighbor-joining phylogenetic tree of sequences from the DGGE profile.

When all operations were complete, biomass samples were obtained from the filter material of each TF. By using the CTO189f/CTO654r primers, the AOB bacterial community was analyzed by PCR–DGGE. The gel is shown in Fig. 5. The 13 bands were identified and excised from the DGGE gel. Sequences of the bands were compared with the available sequences in GenBank, and the phylogenetic tree was constructed (Fig. 6).

The identified species belonged to *Nitrosomonas* (bands A1, A2, A4, A8, and A11), *Nitrosospira* (A9 and A10), *Rhodanobacter* (A6 and A12), *Streptococcus australis* (A13), *Clostridiales* (A3), *Actinomycete* (A7), and an unidentified bacteria (A5). The AOB were divided into the β-subclass and γ-subclass.^23^ There were two genera in the β-subclass: *Nitrosomonas* and *Nitrosospira*. The later included two subgenera: *Nitrosolobus* and *Nitrosovibrio*.^24^ In this study, all identified AOB species belong to the β-subclass, although some identified species were not AOBs, which was due to the specificity of the primers. The specificity of PCR is usually limited by only one AOB specific primer. The CTO189f/CTO654r primers held a higher specificity within the AOB specific primer.^25^ Bacterium for bands A1, A2, A4, and A8 were identified (98%) as *Nitrosomonas* sp. Band A11 was identified (99%) as *Nitrosomonas eutropha*, which has been reported to be a special AOB in aerobic environments and has a capacity for autotrophic denitrification.^26^ Band A9 was 99% similar to an uncultured *Nitrosospira* sp. Band A10 was identified (100%) as *Nitrosospira multiformis*. *Nitrosospira* is a common AOB genus that can compose nitrite reductase and NO oxidoreductase. These enzymes can both catalyze nitrite to N_2_O.^27–29^

## 4. Discussion

### 4.1 Effect of ammonia removal and nitrification by the soil layer

A comparison of the performances of three TFs is presented in Table 2. The STF had a similar NH_4_^+^-N removal rate to that in WTF during periods I and II, but an obviously higher NH_4_^+^-N removal rate in comparison to the WTF during period III. This indicates that the STF (with micron-sized soil) had a better ammonia oxidizing capacity and resistance in comparison to the WTF, especially under a high NH_4_^+^-N loading.

**Table 2:** Removal capacity of pollutant in three TFs

		Period I			Period II			Period III		
		WTF	GTF	STF	WTF	GTF	STF	WTF	GTF	STF
NH_4_^+^-N	Volume loading (g m^−3^ d^−1^)	32.79			85.03			136.35		
	Influent (mg L^−1^)	192.86			550.17			802.08		
	Effluent (mg L^−1^)	29.90	58.24	29.21	83.84	164.14	80.94	400.00	413.58	342.33
	Removal (%)	84.50	69.80	84.86	83.24	67.18	83.82	50.13	48.44	57.32
	Load removal (g m^−3^ d^−1^)	27.71	22.89	27.83	70.78	57.12	71.27	68.35	66.05	78.16
TN	Volume loading (g m^−^3 d^−1^)	33.67			85.64			137.25		
	Influent (mg L^−1^)	198.05			503.76			807.33		
	Effluent (mg L^−1^)	122.63	159.97	120.09	339.33	392.62	292.93	597.02	615.14	517.88
	Removal (%)	38.08	19.23	39.36	32.64	22.06	41.85	26.05	23.81	35.85
	Load removal (g m^−3^ d^−1^)	12.82	6.47	13.25	27.95	18.89	35.84	35.75	32.68	49.20
COD	Volume loading (g m^−3^ d^−1^)	30.76			49.83			86.51		
	Influent (mg L^−1^)	180.92			293.13			508.89		
	Effluent (mg L^−1^)	160.97	117.43	173.86	163.46	170.87	229.53	200.67	202.82	256.68
	Removal (%)	11.03	35.09	3.90	44.24	41.71	21.70	60.57	60.14	49.56
	Load removal (g m^−3^ d^−1^)	3.39	10.79	1.20	22.04	20.78	10.81	52.40	52.03	42.87
Influent COD/TN		0.94			0.59			0.63		
Average effluent NO_2_^−^-N		2.10	11.70	2.50	14.10	21.10	31.00	13.00	32.30	58.60
Average effluent NO_3_^−^-N		90.60	90.00	88.40	241.40	207.30	181.00	169.30	182.20	116.80
Removal COD/TN		0.26	1.67	0.09	0.79	1.10	0.31	1.46	1.59	0.87

In a biochemical system, nitrification is carried out by AOBs, whereby their abundance has a primary impact on the NH_4_^+^-N removal capacity. The abundance of the AOBs was indicated by the brightness of bands A1, A2, and A4 in the lanes of the three TFs, which showed that the abundance of *Nitrosomonas* was similar in the three reactors. However, bands A8, A9, A10, and A11 in the STF somewhat brighter than those of the WTF and much brighter than those of the GTF. The abundance of *Nitrosospira* in the WTF and STF was greater than that in the GTF. This demonstrates that the soil layer in the STF was a more suitable micro-environment for the growth of AOBs, and thus more suitable for increasing their abundance and activity. There were two reasons for the higher NH_4_^+^-N removal and greater abundance of AOBs. The mixed microbes and cellulose formed a biofilm as woodchips were decomposed in the WTF, and the soil layer further extended the appositional growth of the AOBs in the STF. Moreover, the soil layer could impede the transfer of oxygen in the STF, although this increased the ammonia concentration gradient to protect the AOBs in the soil layer against free-ammonia inhabitation, which may have led the better performance of the STF for NH_4_^+^-N removal.

The accumulation of nitrite in the effluents (Table 2) of the STF and WTF was similar in period I (2.5 mg L^−1^ and 2.1 mg L^−1^), whereas this was 31.0 mg L^−1^ and 58.6 mg L^−1^ in the STF during in period II and III, respectively, which were much higher in comparison to the WTF. This demonstrates that there was a much stronger “short-cut” nitrification in the STF. This may have been due to the DO gradient in the soil layer of the STF, which strengthened the anaerobic micro-environment on the surface of the filter material. Band A3 of anaerobic bacteria (*Clostridiales*) in the lane of the STF was brighter than that of the other two TFs, which suggests that the anaerobic micro-environment was relatively higher in the STF. It is known that NOB are more sensitive than AOB to low DO environments,^30,31^ and such a lack of DO could decrease the activity of NOB.

Overall, the STF had a slightly higher ammonia oxidizing capacity in comparison to the WTF; however, the STF also had a much higher nitrite accumulation. This demonstrates that wrapping the soil on to the woodchips did not impact the ammonia oxidization, and “short-cut” nitrification was easily realized.

### 4.2 Enhancement of denitrification by the soil layer

Table 2 shows that the STF had a slightly higher TN removal efficiency (39.4%) in comparison to the WTF (38.1%) during period I. The TN removal rate of the STF was 41.8% and 35.9% during periods II and III, respectively, which were obviously higher than the TN removal rate of the WTF during the same periods (32.6% and 26.1%). This suggests that the addition of the soil layer in the STF improved denitrification. Bio-denitrification has been found to play a major role in TFs, whereas chemical nitrogen removal was determined to be very weak.^21^ Bio-denitrification includes heterotrophic and autotrophic processes.^32,33^

In terms of heterotrophic denitrification, the STF had better anaerobic conditions because the woodchips were wrapped by the soil layer. This could have advanced the decomposition of the woodchips, thus increasing the carbon resources for heterotrophic denitrification, which is supported by the high COD in the effluent of the STF. Moreover, the high accumulation of nitrite in the STF indicates that there should have been a strong “short-cut” nitrification, in which nitrite could directly converted to N_2_ and the carbon resources used for nitrate denitrification could be decreased. This was, therefore, very beneficial for the swine-wastewater digested liquid with a low COD/TN ratio.

Some autotrophic nitrogen removal processes have been observed in wastewater treatment, for example, ANAMMOX. This requires the coexistence of nitrite accumulation and an anaerobic environment in a nitrification reactor.^6,34–36^ The STF in this study satisfied this requirement; hence, ANAMMOX may have occurred to a certain extent. Besides, some autotrophic nitrogen removal has been ascertained, for example, earlier research found that some AOBs could oxidize NH_4_^+^-N to N_2_O in a low DO environment.^26^

## 5. Conclusion

Overall, the addition of a layer of micro-size soil in a TF could increase the ammonia oxidization, nitrite accumulation, and denitrification capacities. By calculating the NH_4_^+^-N and TN removal loads, the NH_4_^+^-N removal rate of the STF during periods I, II, and III was 21.4%, 24.9%, and 18.3% higher, respectively, than that of the GTF. In addition, the TN removal rate of the STF during periods I, II, and III was 104.6%, 89.4%, and 37.5% higher, respectively, than that of the WTF. The abundance of identified AOBs genera, belong to the β-subclass of *Nitrosomonas* and *Nitrosospira*, were enhanced by wrapping a soil layer around the woodchips in the STF. Moreover, the soil layer in the STF provided a better anaerobic micro-environment in comparison to the other TFs, which further strengthened the denitrification process.

## Conflicts of interest

There are no conflicts to declare.

## Supplementary Material
